# Kinetic study of a *β*-mannanase from the *Bacillus licheniformis* HDYM-04 and its decolorization ability of twenty-two structurally different dyes

**DOI:** 10.1186/s40064-016-3496-3

**Published:** 2016-10-21

**Authors:** Jingping Ge, Renpeng Du, Dan Zhao, Gang Song, Man Jin, Wenxiang Ping

**Affiliations:** 1 Key Laboratory of Microbiology, College of Life Science, Heilongjiang University, Harbin, 150080 People’s Republic of China; 2 School of Chemical Engineering and Technology, Tianjin University, Tianjin, 300072 People’s Republic of China

**Keywords:** *β*-Mannanase, *Bacillus licheniformis*, Characterization, Dye decolorization

## Abstract

**Background:**

The microbial *β*-mannanases have been increasingly exploited for bioconversion of biomass materials and various potential industrial applications, such as bleaching of softwood pulps, scouring and desizing, food and feed additive, and oil and textile industries. In this paper, a *β*-mannanase was characterization from the bacteria, *Bacillus licheniformis* HDYM-04, which was a high *β*-mannanase-producing strain (576.16 ± 2.12 U/mL at 48 h during fermentation).

**Methods:**

The michaelis constant (*K*_*m*_) and maximum velocity (*V*_*max*_) of *β*-mannanase were determined. The effect of organic solvents, inhibitors, detergents, chelating agents, oxidizing agents and reducing agents on the stability of enzyme were determined. The degradation of twenty-two structurally different dyes by the purified *β*-mannanase produced by HDYM-04 was determined by full spectrum scan among 200–1000 nm at 0 min and 10 min, respectively.

**Results:**

*β*-Mannanase produced by HDYM-04 was highly specific towards glucomannan, where as exhibited low activity towards guar gum. Michaelis constant (*K*_*m*_) and maximum velocity (*V*_*max*_) of glucomannan substrate were 2.69 mg/ml and 251.41 U/mg, respectively. The activity of different organic solvents showed significantly difference (*p* < 0.05). It retained > 80 % activity in dimethyl sulfoxide, acetone, chloroform, benzene, hexane. In the presence of solvents, citric acid, ethylene diamine teraacetic acid and potassium iodide, it retained > 80 % residual activity. Twenty-two structurally different dyes could be effectively decolourised by *β*-mannanase within 12 h, in which methyl orange (99.89 ± 2.87 %), aniline blue (90.23 ± 2.87 %) and alizalin (83.63 ± 2.89 %) had high decolorization rate.

**Conlusion:**

The obtained results displayed that the *β*-mannanase produced by HDYM-04 showed high stability under different chemical reagents and was found to be capable of decolorizing synthetic dyes with different structures. So, the reported biochemical properties of the purified *β*-mannanase and its rapid decolorizations of dyes suggested that it might be suitable for industrial wastewater bioremediation.

## Background

*β*-Mannanase (endo-1,4-*β*-D-mannanase, EC 3.2.1.78) is a hydrolase that catalyzes the random hydrolysis of *β*-1,4-mannosidic linkages in the main chain of *β*-1,4-D-mannan and releases linear/branched oligosaccharides of various lengths, and it could be classed to the glycosyl hydrolase (GH) families 5 and 26 based on amino acid sequence similarities (Van Zyl et al. 2010; Cantarel et al. 2009). *β*-mannanases have been characterized from a wide range of organisms, including invertebrate, plants, filamentous fungi, yeasts and bacteria. There has been growing interest over the years in the industrial potential of *β*-mannanase degrading enzymes, especially microbial *β*-mannanase (Wang et al. 2010a, b; Chauhan et al. 2012). Microbial *β*-mannanases are the primary endo-type enzymes responsible for degradation of mannan polysaccharides (Liepman et al. 2007; Scheller and Ulvskov 2010). The microbial *β*-mannanases have been increasingly exploited for bioconversion of biomass materials and various potential industrial applications, such as bleaching of softwood pulps, scouring and desizing, food and feed additive, and oil and textile industries due to the various advantages it can act in a wide range of pH and temperature because of which they play important roles in basic research (Dhawan and Kaur 2007; Zhou et al. 2012). So far, various microbial *β*-mannanases from *Streptomyces* sp. (Takahash et al. 1984), *Bacillus subtilis* (Jiang et al. 2006), *Aplysia kurodai* (Zahura et al. 2011), *Bacillus licheniformis* (Songsiriritthigul et al. 2010) and *Trichoderma harzianum* (Ferreira and Ferreira 2004) have been purified and characterized.

Synthetic dyes are classified as anthraquinone, azo, heterocyclic, triphenylmethane (TPM) dyes, and extensively used in several industries including textile, cosmetic, paper, printing, leather-dyeing, pharmaceutical and food industries (Chauhan et al. 2014a, b), but they have caused a serious environmental pollution. Moreover, the exiting dyes usually come from synthetic origin and contribute to more complicate molecular structures making them difficult to biodegrade which most of them are toxic, mutagenic and carcinogenic (Brown and De Vito 1993). The process of dye decolorization based on enzyme is an efficient method and is attracting increasing interest (Erkurt et al. 2007). By means of enzymatic catalyzed oxidative reactions, *β*-mannanase can detoxify phenolic contaminants (Asgher et al. 2008). At present, a lot of studies have focused on microbial enzymes. Certain fungal laccases combined with synthetic or natural mediators have been reported to proved to be suitable tools for textile effluent and dye removal treatments (Kaushik and Malik 2009). Although the *β*-mannanases from *Bacillus* have been already well characterized, there is still absence of information on the enzyme’s kinetic properties and factors that influence stability and the use of *β*-mannanases is still restricted due to high-production costs and low yields (Zhang et al. 2000; Zakaria et al. 1998). Surprisingly, no studies have been implemented to estimate *β*-mannanases from *Bacillus licheniformis* that may take part in the decolorize and biodegrade dyes.

In our previous studies, a *β*-mannanase from *B.licheniformis* HDYM-04 was purified (Ge et al. 2016). However, the stability of chemical reagents and application performances of *β*-mannanase have not been studied. The aim of this study was to carry out preliminary investigation of biocatalytic kinetic properties, stability of organic solvents, including inhibitors, detergents, chelating agents and oxidizing agents and decolorization of multifarious dyes of *β*-mannanase from *B.licheniformis* HDYM-04. Investigation of the application performance of *β*-mannanase would enhance the potential usability in industrial processing.

## Methods

### Microorganism and cultivation

*B.licheniformis* HDYM-04 was isolated from flax-retting water in Bayan County, Heilongjiang Province, P.R. China. This strain was preserved in Key Laboratory of Microbiology, College of Life Science, Heilongjiang University. For the seed culture, one colony was inoculated into 200/250 mL liquid medium (1 % peptone, 0.5 % yeast extract and 1 % NaCl; w/v) and incubated at 37 °C overnight. 2 mL seed liquid of strain HDYM-04 was inoculated into the liquid KGM medium which contained (1 % konjac powder, 1 % peptone, 0.5 % K_2_HPO_4_·3H_2_0, 0.02 % MgSO_4_·7H_2_O, pH 8.0; w/v). The incubation lasted 48 h under the conditions at 37 °C with agitation speed of 160 r/min.

### Protein and enzyme assays

Protein concentration was determined according to the method of Bradford using bovine serum albumin (BSA) as the standard (Bradford 1976). The protein eluted with column chromatography was monitored by taking absorbance at 595 nm. Briefly, 0.1 mL sample was added to 5 mL Comassie Brilliant blue solution (0.1 %, w/v) containing phosphoric acid (85 %, w/v) and mixed. Then, it was allowed to stand at room temperature for 2 min and the absorbance was measured at 595 nm against blind sample which was formed by using pure water instead of enzyme. The *β*-mannanase activity of HDYM-04 was assayed by measuring the amount of reducing sugars released by the enzyme using dinitrosalicylic (DNS) method (Miller 1959). The enzyme assay mixture contained 0.9 ml of 0.5 % (w/v) konjac powder without reducing sugar substrate buffer (0.5 % konjac powder in citric acid-Na_2_HPO_4_ buffer, pH 4.0) and 0.1 mL of appropriately diluted enzyme. The reaction mixture was maintained at 55 °C for 30 min, and then, 3 mL of DNS reagent was added and boiled for 5 min and constanted volume to 25 mL. After cooling to room temperature, the absorbance at 550 nm was measured. One unit of enzyme activity was defined as the amount of enzyme that produced 1 μmol of reducing sugar as a d-mannose standard per minute by 1 mL of enzyme. The crude *β*-mannanase produced by HDYM-04 was obtained according to “Microorganism and cultivation”. The precipitated enzyme was dialysed and monitored at 550 nm followed by activity assay. The crude enzyme was purified to homogeneity by using combination of acetone precipitation, ion-exchange chromatography (DEAE-Cellulose, D3764, Sigma, USA) and gel filtration (Sephadex G-75, Sigma, USA) (Ge et al. 2016).

### Determination of kinetic properties

The michaelis constant (*K*_*m*_) and maximum velocity (*V*_*max*_) of *β*-mannanase produced by HDYM-04 were determined in 0.1 mol/L Tris–HCl buffer (pH 8.0) containing 0.2 − 1 mg/mL substrates (amorophophallus konjac and guar gum), after incubation with 3 mL purified *β*-mannanase produced by HDYM-04 at 60 °C for 10 min, and then, 3 mL of DNS reagent was added and boiled for 5 min and constanted volume to 25 mL. After cooling to room temperature, the absorbance at 550 nm was measured. The data were plotted according to the Lineweaver–Burk method (Zeilinger et al. 1993). Each data was an average of three independent experiments, and every test included three samples.

### Effect of organic solvents on the stability of *β*-mannanase produced by HDYM-04

To determine the effect of organic solvents (dimethyl sulfoxide, ethanol, formaldehyde, acetone, chloroform, benzene, xylene, hexane, petroleum ether) at 50 % concentration on the stability of *β*-mannanase produced by HDYM-04, 1 mL of suitably diluted purified enzyme was mixed with 1 mL of different organic solvents and then incubated at 37 °C for 3 h with constant shaking (150 r/min).

### Effect of inhibitors, detergents, chelating agents, oxidizing agents and reducing agents on *β*-mannanase activity

To study the effects of inhibitors (citric acid, oxalic acid, phenylmethyl sulfonyl fluoride (PMSF), sodium thioglycolate, hydrogen), detergents (cetyl trimethyl ammonium bromide, polyethylene glycol), chelating agents (sodium citrate, ethylene diamine teraacetic acid, sodium azide), oxidizing agents (hydrogen peroxide, ammonium persulfate, potassium iodide), reducing agents (ascorbic acid, dithiothreitol (DTT)) at 1 mM concentrations on the enzyme activity, suitably diluted purified enzyme was preincubated with reagents for 1 h at 37 °C with constant shaking (150 r/min).

### Decolorization of synthetic dyes by the *β*-mannanase produced by HDYM-04

All the tested dyes were purchased from Sigma Company, detailed information was shown in Table 1. The degradation of twenty-two structurally different dyes by the purified *β*-mannanase produced by HDYM-04 was determined by full spectrum scan among 200–1000 nm at 0 min and 10 min, respectively. The decolorization of test dyes were calculated at 37 °C for 6 and 12 h on rotary (160 r/min), respectively. The reaction mixture for the standard assay contained respective dye (0.05 mg/mL) in disodium hydrogen phosphate-citric acid buffer at pH 6.0 and the enzyme solution (5896.4 U/mL) in a total volume of 6 mL. The decolorization rate of dye, expressed as dye decolorization (%), was calculated as the formula: decolorization (%) = (1-A/A_0_) × 100 %, where A_0_: initial absorbance of the dye, A: absorbance of the dye along the time. All experiments were performed in triplicate.

**Table 1:** Characteristics of dyes tested in this work

Dyes	Type	λ_max_ (nm)	Chemical formular
Orange G6	Azo	469	C_16_H_10_N_2_Na_2_O_7_S_2_
Orange I	Azo	467	C_16_H_12_N_2_O_4_S·Na
Methyl orange	Azo	461	C_14_H_15_N_3_NaO_3_S
Ponceau S	Azo	501	C_13_H_9_N_3_NaO_5_
Alizarin yellow R	Azo	373	C_22_H_14_N_6_Na_2_O_9_S_2_
Solvent red 24	Azo	232	C_24_H_20_N_4_O
Amaranth	Azo	508	C_34_H_32_ClN_3_NaO_6_S_2_
Chromotrope 2R	Azo	504	C_16_H_10_N_2_Na_2_O_8_S_2_
Alizarin	Anthraquinone	417	C_14_H_7_NaO_7_S
Methylene blue	Anthraquinone	682	C_16_H_18_N_3_ClS
Fast green3	Triaromatic methane	618	C_37_H_34_N_2_O_10_S_3_Na_2_
Aniline blue	Triaromatic methane	582	C_32_H_25_N_3_Na_2_O_9_S_3_
Coomassie brilliant blue	Triaromatic methane	595	C_45_H_44_N_3_O_7_S_2_Na
Brilliant green	Triaromatic methane	623	C_27_H_34_N_2_O_4_S
Eosin	Triaromatic methane	522	C_20_H_6_Br_4_Na_2_O_5_
Water-soluble melanin	Cyanine	533	C_24_H_19_N_4_
Eosin Y	Three aryl methyl	523	C_36_H_27_N_3_O_5_Br_4_S
Bromothmol blue	Three aryl methyl	420	C_27_H_28_Br_2_O_5_S
Bromophenol blue	Three aryl methyl	422	C_19_H_10_Br_4_O_5_S
5,5′-Dibromo-*o*-cresolsulfonphthalein	Three aryl methyl	648	C_21_H_16_Br_2_O_5_S
Safranine T	Heterocyclic	530	C_20_H_19_N_4_Cl
Neutral red	Heterocyclic	440	C_15_H_17_ClN_4_

### Statistical analysis

All tests were performed in three replications. Average ± standard errors of all obtained date were defined. The average standard errors of the data were expressed. SPSS version 10.0 software (SPSS Inc., Chicago, IL., USA) was used for the statistical analysis; and Tukey test was performed for determining the significant differences at 95 % confidence interval (*p* < 0.05).

## Results

### Kinetic parameters of *β*-mannanase produced by HDYM-04

**Fig. 1:**
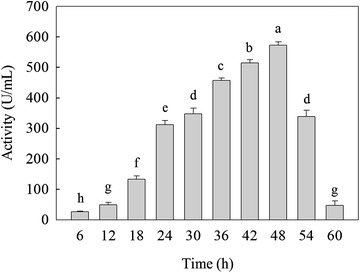
Konjac gum to determine the incubation time of fermentation. *Different letters* represent significant differences (*p* < 0.05) relative to the control

**Fig. 2:**
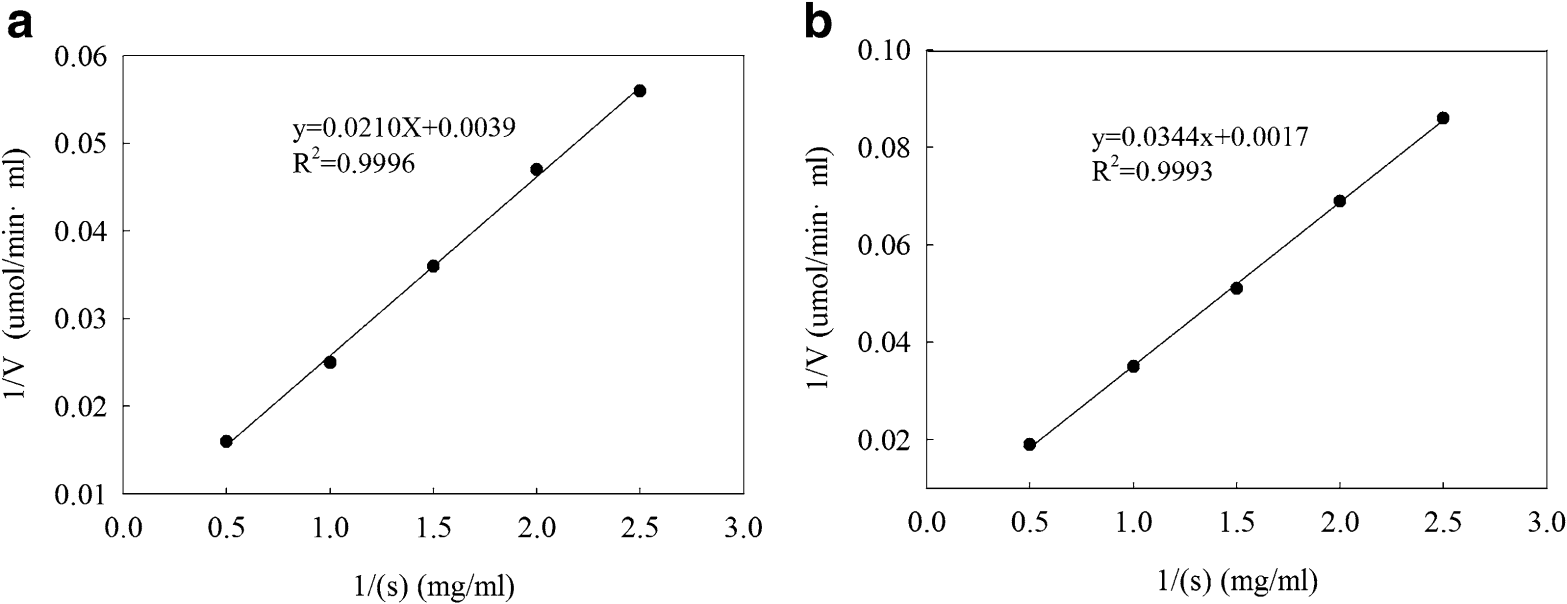
Amorophophallus konjac (**a**) and guar gum (**b**) as the substrate Lineweaver–Burk double bottom. The data were expressed with Lineweaver–Burk plot, and *K*_*m*_ and *V*_*max*_ values were calculated using the nonlinear regression

The *β*-mannanase produced by HDYM-04, which was a high *β*-mannanase-producing strain, and the maximal *β*-mannanase activity was 576.16 ± 2.12 U/mL at 48 h during fermentation (Fig. 1). Michaelis constant could reflect the strength of enzyme substrate affinity. The kinetics (*V*_max_ and *K*_m_) of low viscosity amorophophallus konjac and guar gum hydrolysis by the purified *β*-mannanase produced by HDYM-04 were calculated from Lineweaver–Burk double reciprocal plots (Fig. 2). The *K*_m_ and *V*_max_ values for the purified *β*-mannanase produced by HDYM-04 on amorophophallus konjac and guar gum were 2.69 and 19.26 mg/mL, and 251.41 and 588.24 umol/min mL, respectively. Higher *K*_m_ value of guar gum than amorophophallus konjac suggested the higher affinity of amorophophallus konjac to the purified *β*-mannanase produced by HDYM-04, which was highly in accordance with the result of substrate specificity.

### Effect of organic solvents on the stability of *β*-mannanase

**Fig. 3:**
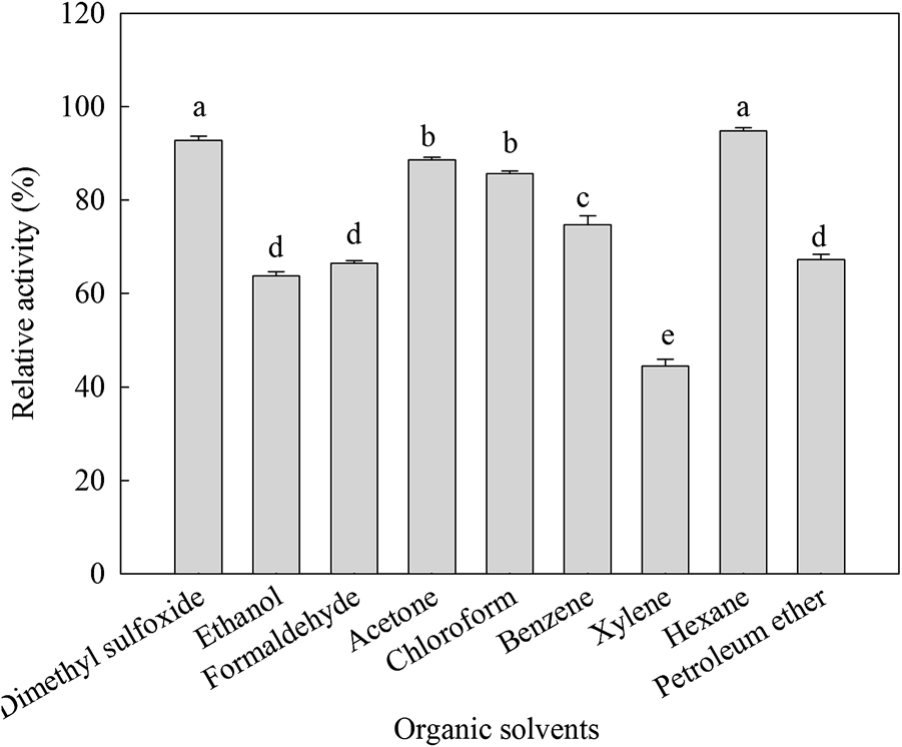
Effect of organic solvents on the activity of purified *β*-mannanase. *Different letters* indicated significant differences among samples incubated with different organic solvents (*p* < 0.05)

The effects of organic solvents on the *β*-mannanase activity produced by HDYM-04 are shown in Fig. 3. In the present study, *β*-mannanase produced by HDYM-04 remined stable after 3 h of preincubation with most of the tested organic solvents. The activity of different organic solvents showed significantly difference (*p* < 0.05). It retained > 80 % activity in dimethyl sulfoxide, acetone, chloroform, benzene, hexane. The enzyme activity significantly higher in dimethyl sulfoxide (93.4 ± 1.74 %) and hexane (94.34 ± 1.19 %) compared to that in other organic solvents (*p* < 0.05). Furthermore, ethanol induced decrease of the enzyme activity to 63.21 ± 2.05 %, and xylene, which is a strong reducing agent on disulphide bonds, strongly inhibited the enzyme to 43.33 ± 1.53 %.

### Effect of inhibitors, detergents, chelating agents, oxidizing agents and reducing agents on *β*-mannanase activity

The *β*-mannanase produced by HDYM-04 was found to be stable with most of the tested agents (Table 2). The inhibition of different reagents showed significantly difference (*p* < 0.05). In the presence of solvents; citric acid, ethylene diamine teraacetic acid and potassium iodide, it retained > 80 % residual activity. Furthermore, phenylmethyl sulfonyl fluoride and dithiothreitol induced decrease of the *β*-mannanase activity to 26.98 ± 2.44 and 27.63 ± 2.41 %, and cetyl trimethyl ammonium bromide, which is a strong detergents on disulphide bonds, strongly inhibited the enzyme to 14.15 ± 1.21 %. However, in the presence of solvents polyethylene glycol and sodium citrate, *β*-mannanase activity decreased to 79.62 ± 2.47 and 78.87 ± 3.23 %, respectively.

**Table 2:** Effect of inhibitors, detergents, chelating agents, oxidizing agents and reducing agents on the activity of purified *β*-mannanase

Reagent	Relative activity (%)
Control	100a
Inhibitors	
Citric acid	87.53 ± 2.34b
Oxalic acid	68.76 ± 1.98c
Sodium thioglycolate	45.34 ± 2.67d
Hydrogen	37.29 ± 1.34de
Phenylmethyl sulfonyl fluoride	26.98 ± 2.44e
Detergents	
Polyethylene glycol	79.62 ± 2.47b
Cetyl trimethyl ammonium bromide	14.15 ± 1.21f
Chelating agents	
Ethylene diamine teraacetic acid	82.43 ± 1.23b
Sodium citrate	78.87 ± 3.23b
Sodium azide	65.98 ± 1.34c
Oxidizing agents	
Potassium iodide	83.23 ± 1.23b
Ammonium persulfate	66.37 ± 1.19c
Hydrogen peroxide	45.82 ± 2.12d
Reducing agents	
Ascorbic acid	38.81 ± 1.13de
Dithiothreitol	27.63 ± 2.41e

The 100 % activity represented the control enzyme activity without any agents. Different letters represent significant differences (*p* < 0.05) relative to the control

### Decolorization of synthetic dyes

**Fig. 4:**
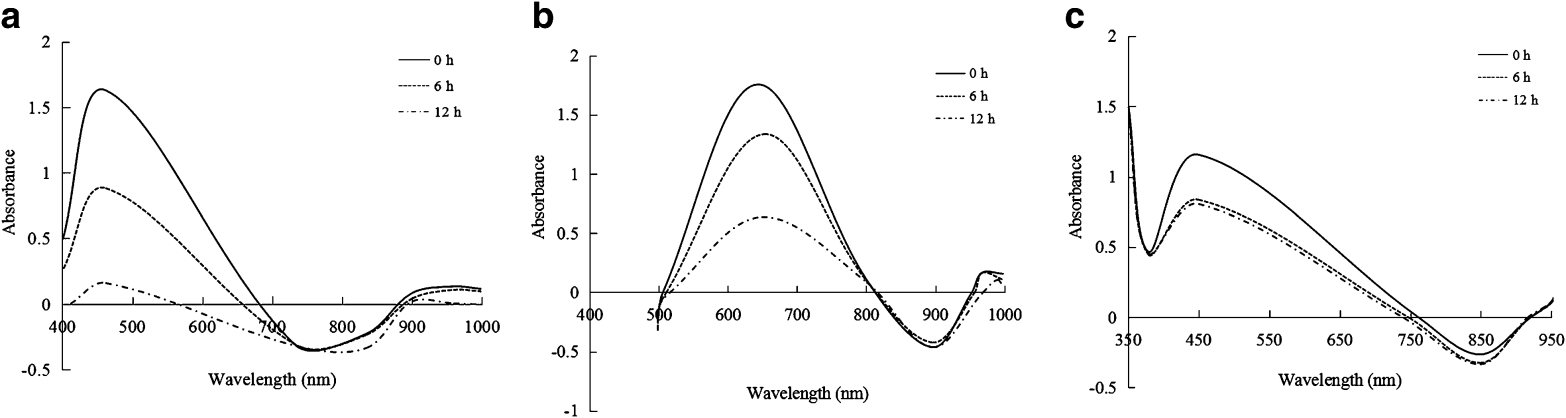
Degradation of dyes by purified *β*-mannanase produced by HDYM-04 (**a** methyl orange, **b** aniline blue, **c** alizalin)

The decolorization of various dyes with different structural patterns were investigated by using purified *β*-mannanase produced by HDYM-04. Our system was able to efficiently degrade a number of commercial textile dyes. Table 3 showed the degradation of twenty-two structures of different dyes including azo, anthraquinone, arylmethyl and other structures of dyes by the purified *β*-mannanase from HDYM-04. The best decolorization overall (80-100 %) were obtained with reactive methyl orange, aniline blue and alizalin within 12 h (Fig. 4). The remaining nineteen dyes were degraded on different extend within 12 h as revealed. Somewhat lower decolorization (30–70 %) was obtained with basic violet 3, ponceau S, water-soluble melanin, coomassie brilliant blue and brilliant green. This could be due to enzyme inhibition (by some products generated in the decolorization process) or substrate inhibition. However, the eosin, amaranth, chromotrope 2R, alizarin yellow R, methylene blue, fast green 3 and neutral red were nearly not decolorized by the purified *β*-mannanase from HDYM-04, which was probably due to the complexity of dye structures.

**Table 3:** Decolourisation of dyes by *β*-mannanase produced by HDYM-04

Dyes	Decolourisation (%)	
	6 h	12 h
Methyl orange	54.25 ± 2.34b	99.04 ± 0.03a
Aniline blue	41.11 ± 2.01e	90.23 ± 2.87b
Alizarin	23.86 ± 2.12e	83.63 ± 2.89c
Water-soluble melanin	64.83 ± 2.56a	68.13 ± 3.56d
Ponceau S	11.72 ± 1.55f	60.49 ± 3.88d
Brilliant green	6.57 ± 0.56g	34.46 ± 2.32e
Coomassie brilliant blue	16.40 ± 2.31f	30.74 ± 2.37e
Bromophenol blue	11.40 ± 1.76f	27.52 ± 2.34e
Bromothmol blue	5.52 ± 1.22g	27.14 ± 2.87e
Solvent Red 24	11.12 ± 1.87f	23.28 ± 2.23ef
Orange G6	3.08 ± 0.23g	19.63 ± 1.07f
Orange I	5.67 ± 0.78g	18.15 ± 0.11f
Safranine T	4.34 ± 0.76g	17.39 ± 2.82f
5,5′-Dibromo-*o*- cresolsulfonphthalein	11.11 ± 1.87f	16.33 ± 2.11f
Eosin Y	14.78 ± 2.11f	16.19 ± 1.76f
Neutral red	0 ± 0 h	7.31 ± 0.87g
Methylene blue	4.18 ± 0.23g	4.29 ± 0.28g
Fast Green3	2.94 ± 0.18g	2.94 ± 0.29gh
Amaranth	0.29 ± 0.14h	1.25 ± 0.11h
Chromotrope 2R	0.46 ± 0.04h	0.68 ± 0.09h
Eosin	0.29 ± 0.01h	0.29 ± 0.03h
Alizarin yellow R	0 ± 0h	0 ± 0h

The different letters in the same column of the data indicate the level of significant differences at *p* < 0.05

## Discussion

In conclusion, this study reported the some characterization of a *β*-mannanase produced by HDYM-04. The property of enzyme to maintain a high production at a short time was interesting from the point of view of industry. To the best of our knowledge, this was the first report about the production of *β*-mannanase from HDYM-04 at 37 °C. Similarly, *Paenibacillus* sp. DZ23 and *B.subtilis* NM-39 produced enzyme at 37 °C with glucomannan and locust bean gum as the substrate (Chandra et al. 2011; Mendoza et al. 1994). Other *B.subtilis* strains separated so far from producing maximum enzyme at below 50 °C (Zhou et al. 2012). Some *B.subtilis* strains can produced enzyme at up to 45 °C (Khanongnuch et al. 1998). The purified *β*-mannanase produced by HDYM-04 shows higher affinity toward glucomannan substrate (*K*_*m*_ and *V*_*max*_ were 2.69 mg/mL and 251.41 U/mg, respectively) than that of other mannans like guar gum. But, the result was higher than that *β*-mannanase of *Paenibacillus* sp. DZ3 (*K*_m_ 1.05 mg/mL) to amorophophallus konjac (Mendoza et al. 1994), and *β*-mannanase of *B.nealsonii* PN-11 (*K*_m_ 11.59 mg/mL) to guar gum (Chauhan et al. 2014a, b). Kinetic studies revealed that the enzyme had more affinity toward natural glucomannan, and hence it was applicable in the food industry for the production of oligosaccharides. In the present study, the *β*-mannanase retained > 80 % activity in dimethyl sulfoxide, acetone, chloroform, benzene, hexane. This could be due to well-known fact that hydrophilic solvents are usually superior to hydrophobic solvents for the better enzyme activity, as the earlier have a greater tendency to bind water tightly, which is essential for catalytic activity. Similar results were observed with mannanase from *B. subtilis* G1 which showed 11–53 % reduction in enzyme activity by the addition of organic solvents (Vu et al. 2012). In the presence of solvents; citric acid, ethylene diamine teracetic acid and potassium iodide, it retained > 80 % residual activity. The strong inhibitory effect of cetyl trimethyl ammonium bromide, a potent cation surfactant, could be due to the destruction of the conformation of mannanase.

Besides, we have found that the *β*-mannanase produced by HDYM-04 we used show remarkably high activity and found to be capable of decolorizing and degrading different structures of synthetic dyes. Though lots of papers reported the degradation and decolourisation of synthetic dyes by other enzymes, some studies demonstrating that laccases from *Leptomitus lacteus* could made different dyes decolorization (Svobodová et al. 2008). Such as, *B.cereus* could decolorize 85 % of an azo dye for 120 h of incubation (Kanagaraj et al. 2012) and a novel laccase from *B.subtilis* WD23 could decolorize 50-90 % of congo red and methyl orange, which suggested the potential application of spore laccase in dyestuff treatment (Wang et al. 2010a, b). Unfortunately, the majority of dyes are chemically stable and still resistant to microbiological attack. The differences in the decoloriaztion efficiencies may be attributed to the various chemical structures of different dyes. The most employed dyes belong to the azo and triaromatic class which accounts for the 80 % of all textile dye produced. The best decolorization over was obtained with reactive conge red methyl orange and titan yellow. *β*-mannanase may be modify azo dye structures by destroying their chromophoric assemblies, phenoxyl radicals are generated in the reaction course (Muralikrishna et al. 1995). Compared with the *β*-mannanase produced by HDYM-04, these enzyme decolorization efficiency were relatively low. The *β*-mannanases are oxidase that cataluze oxidation reactions and hydrolysis reactions in many phenolic and organic substrates coupling with reduction reactions that transfer molecular oxygen to water (Murugesan et al. 2007). Triaromatic methane dyes represent an especially recalcitrant class of compounds. The present study confirms the ability of methyl orange and aniline blue purified *β*-mannanase to decolorize amaranth, chromotrope 2R, amaranth and eosin with decolorization efficiency of more than 90 % in short time. This could be due to the presense of electron donating methy and methoxy groups on the triaromatic methane dyes. The results obtained in this study were in agreement with results reported previrously for *Pleurotusostreatus* laccase (Kumar et al. 2012) and *P.variabile* laccase (Forootanfar et al. 2011). They also demonstrated that different decolorization rates were attributed to the specific catalytic properties of the individual enzymes and to the structure of dyes. To our knowledge, this is the first description of a bacterial *β*-mannanase from HDYM-04 able to degrade different dyes. Furthermore, the decolorization of dyes by *β*-mannanase produced by HDYM-04 is simple and cheap. So, the broad substrates specificity of enzyme rendered its great potentials in industrial applications, such as degradation of dyes from acidic textile effluents and the purified *β*-mannanase produced by HDYM-04 could be successfully employed for the treatment of dyes bearing industrial wastewater as it had prominent capacity to degrade other different dyes. Some studies found that, the degradation of azo dyes could result in the production of compounds of increased toxicity. However, most studies failed to evaluate the toxicity of either the dyes and/or the reaction products (Gottlieb et al. 2003). So, to further investigate its effect on the toxicity of dye decolorization, the toxicity experiment will be carry out.

## Conclusions

To sum up, the purified *β*-mannanase produced by HDYM-04 showed higher affinity toward glucomannan substrate (*K*_*m*_ and *V*_*max*_ were 2.69 mg/ml and 251.41 U/mg, respectively) than that of other mannans like guar gum. The enzyme obtained from this research possessed much higher stability in inhibitors, detergents, chelating agents, oxidizing agents and reducing agents. Furthermore, this enzyme could resist citric acid, ethylene diamine teraacetic acid and potassium iodide with more than 80 % maximum activity remained. Besides, this study represented the first attempt to decolorize the mixtures of dyes by purified *β*-mannanase from HDYM-04. Thus, the *β*-mannanase has been successfully identified and, from this study, it has good potential in applying to decolorize dyes in textile wastewaters, particularly for water recycling. Further studies should be attempted to evaluate their feasibility in industrial uses.
